# Male reproductive tactics in house mice: Consistent individual differences, intrinsic factors and density effects

**DOI:** 10.1111/1365-2656.70039

**Published:** 2025-04-12

**Authors:** Fragkiskos Darmis, Alexandros Vezyrakis, Anja Guenther

**Affiliations:** ^1^ Research Group Behavioural Ecology of Individual Differences Max Planck Institute for Evolutionary Biology Plön Germany; ^2^ Department of Animal Ecology, Institute for Biochemistry and Biology University of Potsdam Potsdam Germany

**Keywords:** alternative reproductive tactics, male–male competition, mammals, multi‐state modelling, roamers, sexual selection, status‐dependent selection, territorials

## Abstract

1. Alternative reproductive tactics (ARTs) describe non‐reversible or flexible alternative strategies that secure fertilization. For example, some male defend territories with females while others attempt sneaky matings. Often, ARTs are considered to be status‐dependent and are explained by differences in mass or competitive ability. However, most studies on ARTs only approximate their fitness effect, ignore males that never reproduced and consider status (e.g. weight) as the sole mediator of ARTs.
2. We used 244 male mice, *Mus musculus domesticus*, from semi‐natural populations, to describe ARTs in *Mus Musculus* for the first time. We followed males throughout their life and categorized them as territorials or roamers over multiple monthly intervals, after validating our method of assigning tactics with detailed spatial data.
3. We explored if tactic choice is repeatable, whether multiple social and/or intrinsic factors predict tactic choice and transitions between tactics, and tested for fitness and physiological differences between ARTs.
4. Tactic choice was repeatable, but males switched flexibly between tactics. Tactic choice was associated with mass, age, the operational sex ratio and population size. Territorials had a higher probability of reproduction, but a lower gonadosomatic index.
5. Our results reveal a personality component of ARTs, confirm equal mean fitness among tactics and suggest tactic choice as a multifaceted decision under various selective pressures.

## Graphical abstract

This study investigates alternative reproductive tactics (ARTs) in male house mice and establishes a comprehensive framework for their maintenance. It provides new insights into the expression of ARTs and the factors influencing male reproductive success and survival, with implications for adaptation in changing environments.

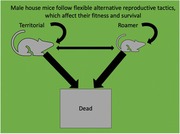

## Οι εναλλαντικές τακτικές αναπαραγωγής επηρεάζουν όλο το φάσμα της ζωής των ζώων

1. Οι εναλλακτικές τακτικές αναπαραγωγής (ΕΤΑ‐Alternative reproductive tactics) περιγράφουν τις διαφορετικές στρατηγικές τις οποίες χρησιμοποιούν άτομα του ίδιου γένους μέσα σε έναν πληθυσμό για την αναπαραγωγή τους. Για παράδειγμα, κάποια αρσενικά υπερασπίζονται και «μονοπωλούν» ομάδες θηλυκών, ενώ άλλα προσπαθούν να αναπαραχθούν με κάποια από τα θηλυκά όταν βρουν ευκαιρίες. ∑υχνά, οι ΕΤΑ εξηγούνται μόνο με βάση το βάρος και τη δύναμη, διότι τα βαρύτερα και δυνατότερα αρσενικά είναι αυτά που μπορούν να ελέγχουν τις ομάδες θηλυκών.
2. ∑ε αυτή τη μελέτη, επικεντρωνόμαστε σε οικιακούς ποντικούς, *Mus musculus domesticus*, οι οποίοι ζουν σε σπίτια και σιταποθήκες. Για πρώτη φορά, καταγράφουμε δύο τακτικές σε αρσενικά αυτού του είδους: κάποια μονοπωλούν και μένουν σε περιοχές με θηλυκά και παράλληλα αποκλείουν την πρόσβαση άλλων αρσενικών στην περιοχή τους (εδαφικά/territorials) και αναγκάζουν αυτά τα αρσενικά να περιπλανώναι (περιπλανώμενα/roamers) και να προσπαθούν να αναπαραχθούν ψάχνοντας θηλυκά τυχαία. Επίσης, αυτές οι τακτικές είναι ευέλικτες (flexible), δηλαδή το ίδιο αρσενικό μπορεί να αλλάζει μεταξύ των δύο κατά τη διάρκεια της ζωής του. Αυτές οι αλλαγές εξηγούνται από τις αλληλεπριδράσεις μεταξύ των ακόλουθων μεταβλητών:
  - Από το βάρος και την ηλικία, με τα βαρύτερα/μεγαλύτερα σε μάζα αρσενικά να είναι εδαφικά γιατί μπορούν να ανταγωνίζονται άλλα αρσενικά; και τα γηραιότερα να περιπλανώνται διότι με την πάροδο του χρόνου χάνουν αυτήν την ικανότητα.
  - Από κοινωνικούς παράγοντες, όπως το πόσα θηλυκά υπάρχουν στον πληθυσμό; διότι όταν δεν υπάρχουν πολλά ακόμα και τα εδαφικά αρσενικά αναγκάζονται να τα αναζητήσουν.
  - Από διαφορές στη φυσιολογία μεταξύ των αρσενικών.
3. Αυτές οι αλλαγές εξηγούνται επίσης από την προσωπικότητα (personality) των ποντικιών; με άλλα λόγια κάποια αρσενικά συνεχώς ακολουθούν μία διαφορετική τακτική σε σχέση με τα υπόλοιπα του πληθυσμού. Τέλος, οι τακτικές παρουσίασαν *ισοδύναμη αρμοστικότητα/εξελικτική αναπαραγωγική επιτυχία*, αλλά τα εδαφικά αρσενικά είχαν μεγαλύτερη πιθανότητα να αναπαραχθούν τουλάχιστον μία φορά. Αυτό υποδηλώνει ότι η διαδικασία λήψης αποφάσεων κατά την αναπαραγωγή βρίσκεται υπό διαφορετικές εξελικτικές πιέσεις στους αρσενικούς οικιακούς ποντικούς. Θεωρούμε ότι η δυνατότητα προσαρμογής των ποντικιών (και άλλων ζώων) σε αλλαγές του φυσικού τους περιβάλλοντος, για παράδειγμα λόγω της κλιματικής αλλαγής, θα είναι διαφορετική ανάλογα με την τακτική που ακολουθούν.

## 1 INTRODUCTION

In many species, males use alternative reproductive tactics (i.e. discrete behavioural/morphological/physiological traits; ARTs) to maximize their fitness (Brockmann, 2001; Brockmann & Taborsky, 2008; Dougherty et al., 2022; Gross, 1996; Kustra & Alonzo, 2020; Schradin & Lindholm, 2011; Stockley et al., 1994; Taborsky et al., 2008). For example, in the Alpine chamois, *Rupicapra rupicapra*, some males defend areas from intruders (i.e. territory holders), while others (nonterritorial) attempt trespassing on other males' territories (Corlatti et al., 2012; Taborsky et al., 2008). ARTs can be non‐reversible, genetically encoded or flexible (reviewed in Gross, 1996), and evolve if there are fitness benefits associated with pursuing an alternative (Taborsky et al., 2008). For example, male striped mice (*Rhabdomys pumilio*) can switch from territorial to roamer (Schradin & Lindholm, 2011). Phenotypically flexible ARTs are also described in other rodents, such as prairie voles (*Microtus ochrogaster*, Shuster et al., 2019) or ground squirrels (*Urocitellus columbianus*, Balmer et al., 2019).

In general, ARTs are more frequently detected in males due to greater variability in male reproductive success (Taborsky et al., 2008) and the higher male investment in reproductive traits that can be exploited by conspecifics that pursue an alternative tactic (Clutton‐Brock, 2007, 2009; Queller, 1997; Taborsky et al., 2008; Taborsky & Brockmann, 2010). Flexible tactics (common in fish, Karkarey et al., 2017; Taborsky & Brockmann, 2010) can evolve due to instability in spatial and temporal mate availability (Brockmann & Taborsky, 2008; Wolff, 2008), due to competition or predation risk. For example, elevated female densities or a male‐biased operational sex ratio (OSR) favour roaming among female groups, and low population densities or a female‐biased OSR favour female monopolization (Wolff, 2008), as shown in male musk oxen *Ovibos moschatus* (Forchhammer & Boomsma, 1998; Wolff, 2008).

The differential expression of male ARTs is considered to be condition‐ or status‐dependent (Brockmann, 2001; Gross, 1996; Johnson & Brockmann, 2012; Schradin & Lindholm, 2011; Shuster & Wade, 2003) or dependent upon female activity (Buzatto & Machado, 2008; Schradin & Lindholm, 2011; Stockley et al., 1994; Taborsky et al., 2008), population size (Radwan, 1993; Radwan et al., 2014) and density (Karkarey et al., 2017; Wolff, 2008) or local ecology (e.g. Monroe et al., 2016). In general, individual competitive ability is often a good proxy of tactic choice for condition‐ and/or status‐dependency (Stockley et al., 1994), from arthropods (Brockmann, 2001; Hunt & Simmons, 2001; Perdigón Ferreira & Lüpold, 2022) to fish (Taborsky, 2008) and primates (Setchell, 2008). Overall, ARTs consist of discrete allocation strategies among conspecifics: some individuals (usually males) directly access heterosexuals (e.g. secondary sexual traits or harem defence) and others exploit conspecifics' effort and get direct access to gametes (‘bourgeois’ and ‘parasitic’ tactics; Taborsky, 1997), that is an alternative ‘best of a bad job’ (Dawkins, 1980) tactic of opportunistic matings.

In the present study, we explored if male house mice (*Mus musculus domesticus*) follow ARTs and if multiple social and intrinsic factors can affect the expression and transition between tactics at the individual level. House mice provide an excellent model to study ARTs in the context of reproductive competition. First, condition‐ and density‐dependent alternative breeding tactics have been established in females, which use a combination of solitary and communal breeding to raise litters (Ferrari et al., 2019, 2022). However, male ARTs have never been described before in males despite adult male aggressive interactions for territorial acquisition (Gray & Hurst, 1997), access to resources, mating opportunities or protection from predators and environmental conditions (Kaufmann, 1983; Ord, 2021). Consequently, these behaviours might lead conspecifics to wander around, possibly attempting sneaky matings. Moreover, multiple paternity ratios go up to ~40% in mice (Schradin et al., 2010)—on average, 21% of litters were multiple‐sired (Porwal et al., 2023)—a number comparatively high compared to other rodents (reviewed in Solomon & Keane, 2008) and other mammals in general (reviewed in Cohas & Allainé, 2009). This means that territorial mice lose paternity and implies that males following an alternative reproductive strategy (than territorial acquisition) might exist and steal paternity.

Here, we quantified male tactics (roamer & territorial) across two generations of semi‐naturally living male house mice in four replicated populations and up for to 11 months per individual (*N* = 244). We also measured the growth and reproduction of these males in their adult life. We then compared two methods to assess male ARTs: (1) we quantified where males were caught per month (inside a nest‐box—termed territorials hereafter—or roaming around) and (2) contrasted those data to more detailed RFID‐ antenna data (from two independent populations) quantifying males' location for 2 months continuously (König et al., 2015). Using these data we checked if male tactic was repeatable, something that to our knowledge has never been tested before in mammals in the context of ARTs by also having all males under monitoring (for similar long‐term studies that quantified ARTs see Balmer et al., 2019; Fasel et al., 2016). Importantly, we expected males to show plastic tactics since environmental and social conditions vary continuously, spatially and temporally, in house mice populations (DeLong, 1967; Singleton et al., 2005; Wilson et al., 2018). Next, we explored if ARTs yield different fitness (Brockmann & Taborsky, 2008; Gross, 1996) and quantified aspects of males' social environment (OSR and population size), as well as intrinsic factors such as mass or age, to test their correlation with the choice of ARTs. We predicted tactic choice to depend on individual mass since heavier males should be stronger and have increased competitive abilities (Schradin, 2019), thus be territorial. Then, we expected tactic choice to depend on age, with the probability of territoriality increasing when males grow strong but then gradually dropping because of a decreased ability to outcompete conspecifics as males become older; and on the number of receptive females, which should drive whether males defend or roam for mating opportunities. Last, we expected ARTs to correlate with population size because an increasing population inevitably results in a quicker depletion of available nest‐boxes (or more generally territories; explained in Section 2.1), and therefore should predict a higher probability of roaming. We also modelled the probability to transition between territoriality to roaming and vice versa, as well as the hazard ratios of each tactic, using a multi‐state model. Here, we specified that transitioning from territoriality to roaming, territoriality to death and roaming to death depend on the intensity of intrasexual competition, available territories and the age of individuals: higher competition (increased OSR or population size) should lead to higher injury or death rates, and older males should have decreased competitive ability and thus be more prone to displacement from territories or to die (from either tactic). Additionally, we expected age, the rate of male–male competition and population size to affect the transition from roaming to territoriality if young roamers that grow older become more competitive and if less individuals and competitors (other males) in the population increase the opportunities for territorial acquisition. Last, we explored if different ARTs were linked to physiological differences such as sperm count, body length and testis to body mass ratio because theory predicts roamers to experience an increased risk of sperm competition and thus invest more in sperm traits (Dougherty et al., 2022; Kustra & Alonzo, 2020).

## 2 METHODS

### 2.1 Animals and housing

Mice descended from wild populations sampled in the Cologne/Bonn region of Germany (*N* = 18 original breeding pairs; 50°45′ N–51° N, 6°45′ E–7° E). Founding mice (*N* = 160, 80 males, 80 females) for the semi‐natural populations were distributed to four replicate enclosures (19.6 m^2^ each), with 20 males and 20 females founding each population.

For house mice, the natural environment is a barn or a human shelter (König & Lindholm, 2012) and, to mimic such an environment, each semi‐natural enclosure was equipped with various nesting materials and 12 nest‐boxes. These nest‐boxes serve as the main location for mouse families to live, breed and raise offspring and are the core range of a home range which is actively defended by the adult (i.e. territory holder) male (Figure S1). While home ranges are usually larger (Figure S1), the nest‐boxes are actively defended against intruders. Food and water were provided ad libitum and distributed uniformly across the enclosure at nine feeding stations. Such conditions are similar to those found in natural European populations of house mice (Anderson, 1982).

We monitored population developments every 4–5 weeks. Population development and densities in semi‐natural enclosures mimic mice populations that are under long‐term observation with the possibility for dispersal (König & Lindholm, 2012). During these monitorings, we caught all mice within an enclosure, measured body mass, checked for fresh bite marks and took a tissue sample of new individuals (weighing >10 g) for parentage assignment. New animals (>10 g) received an RFID PIT tag (Planet ID, 1.4 × 9 mm) for permanent recognition. Whenever a given population reached at least 80 chipped offspring, we removed the older generation (usually after 8–10 months) to keep the density below the carrying capacity. For this reason, our observations are restricted to a maximum age of 11 months. Some of the removed animals were euthanized in order to obtain physiological characteristics, for example conduct sperm counts.

### 2.2 Reproductive tactic assignment

During our regular monthly population monitorings, each adult male was assigned a tactic based on its location (a visualization in Figure S2). When entering the enclosure, some mice (all identifiable through their chip) were inside nest‐boxes or entered one, while others remained outside either because they did not attempt to enter a nest‐box or because they were chased out immediately when attempting to do so. Nest‐boxes were then closed (i.e. the identity of animals inside the nest‐box is known) and all animals (inside nest‐boxes and all animals outside) located in the enclosure were removed, identified and weighed (to the closest decimal, e.g. 28.4 g). Since we could individually identify all mice, we could infer survival based on the presence/absence of animals; thus, we also have monthly estimates for population density and individual characteristics such as survival.

**FIGURE 1:**
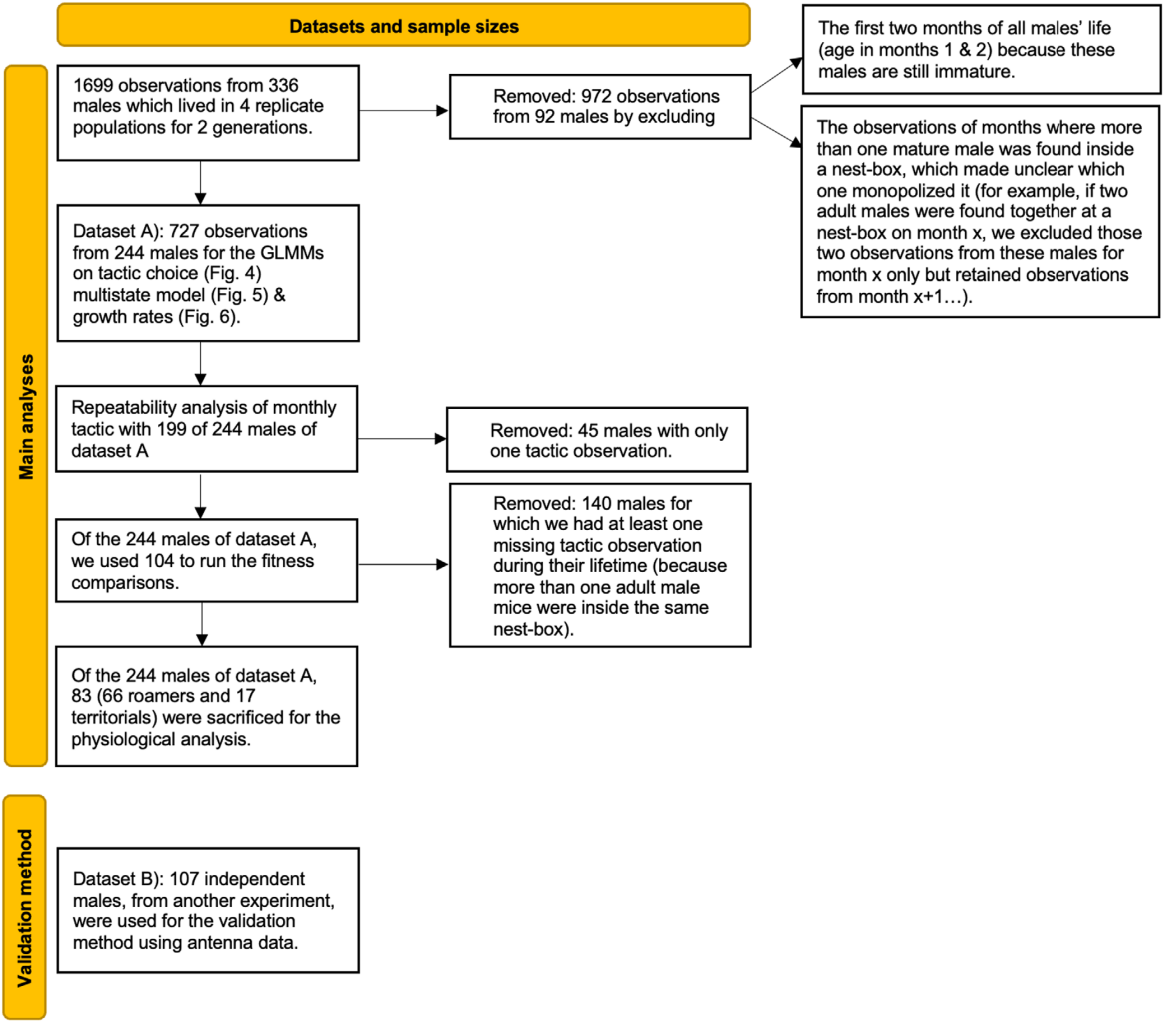
The datasets and sample sizes used in this study.

**FIGURE 2:**
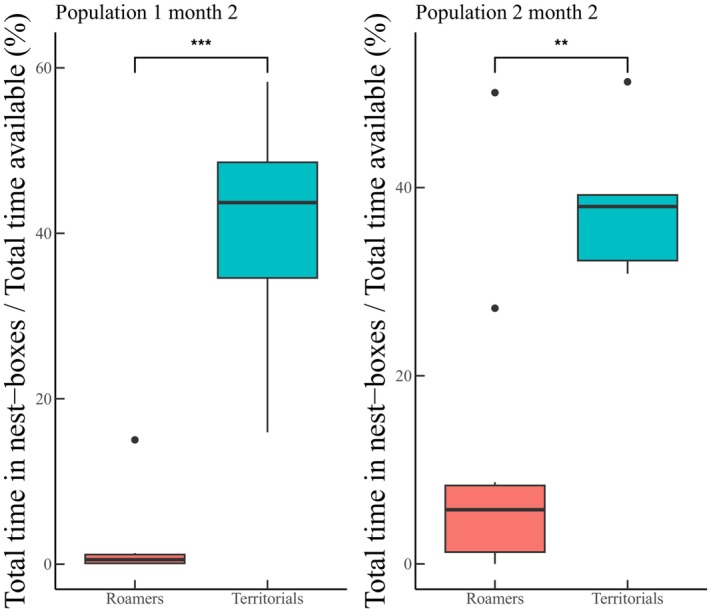
Validation of tactic assignment of monitoring data from antenna data (population 1: 8 roamers & 6 territorials; population 2: 10 roamers & 5 territorials). A comparison of the total time roamers and territorials (assigned during monitoring) spent inside nest‐boxes within a month. The *y*‐axis plots the percentage of time (in seconds) territorials or roamers (the tactic of each male was based on its position during a monitoring; inside a nest‐box or outside) spent inside nest‐boxes over the total time available per month (we divided the individual seconds spent inside all nest‐boxes to the total seconds available between our two monitorings and we averaged this number for all individuals per tactic). The second month is used since during the first males are expected to adjust, explore and then follow a tactic.

If an adult male was located inside a nest‐box as the only adult male there, it was identified as the territory holder, and if outside a nest‐box, as a roamer. We excluded observations from months when more than one mature (>4 weeks) male was found at a particular nest‐box as it was unclear which male monopolized the nest‐box (but these data accounted for a small proportion of the initial sample size: ~10%; *N* = 199/1699; see Figure 1). To validate that this ‘snapshot’ of male locations during the monitorings actually reflects meaningful differences in male behaviour throughout time, we ran an additional experiment with 107 males for 2 months in two additional populations (these populations were used only to validate our tactic assignment and males were not part of the 244 individuals used in all other analyses; see Figure 1). During this validation experiment, we fitted the entrances of all nest‐boxes with RFID antennas to record the locations and durations of stays within a nest‐box. We calculated the amount of time each adult male spent within a nest‐box and correlated this with the tactic assignment during the monitorings (Figure 2).

### 2.3 Datasets

We used two independent datasets, as displayed below (Figure 1): one for validating our tactic assignment as a biologically meaningful method for assigning tactic; and another one for all other analyses of ARTs.

### 2.4 Fitness

We used 17 microsatellite markers to determine parentage and assign fitness using the procedure adapted from Linnenbrink et al. (2013). DNA was extracted from ear clips, amplified using a Multiplex PCR kit (QIAGEN) and the samples were run on an ABI 3730 Sequencer (Applied Biosystems). We used GeneMarker (V2.6.4) to identify alleles and Colony [©COLONY Zoological Society of London (ZSL)] to assign the parentages based on the maximum likelihood of each potential parental pair. We conditioned our parentage assignment on the following assumptions: sexual reproduction, polygamous mating system, possible inbreeding, and all animals being present the month before sampling (i.e. when juveniles were born) as possible parents. Overall, our fitness estimates quantify the number of offspring that survived to at least 10 g (Figure S4).

To explore differences between ARTs in reproductive success we quantified fitness in four different ways for 104 males (50 that reproduced and 54 that never reproduced). We focused on 104 (from the 244) males for which we could assign their tactic choice every month without ambiguity and for which we had all monthly tactic observations throughout their life. In other words, we excluded 140 males of which at least one tactic assignment was uncertain (explained in Figure 1). By doing so, we reduced noise for our predictor of fitness [formula (1)]. We quantified fitness using: (1) a binary (0/1) variable describing successful or not reproduction; (2) the total number of offspring of each male (absolute fitness); (3) the number of offspring each male produced during its life divided by the mean number of offspring of all males in that semi‐natural enclosure (relative fitness); (4) the total number of offspring of each male divided by its lifespan (defined as fitness/age in the *Modelling* part). Metric 3 identifies the relative fitness of each male based on the tactic each male followed throughout its life. Metric 4 ‘stratifies’ individuals based on both their offspring yield and their survival. We used individuals that reproduced successfully, along with those that did not, as omitting the latter would inevitably increase average estimates of fitness as well as decrease variance of fitness for each tactic (Shuster, 2011).

We used the following formula as a predictor (in the fixed effects part of the formula) in all analyses, which had fitness as a response:

(1) $$\frac{R}{R+T},$$

where R means roamer and T territorial. This metric, which measures the ratio of the times each male was caught as a roamer to the total times he has been caught (since a male can be caught as either territorial or roamer), allowed us to account for the individual lifetime tactic decisions.

### 2.5 Correlates and transitions rates of ARTs

Considering the correlations between ARTs with intrinsic or environmental aspects, as well as the transition probabilities between the tactics, we first used the OSR, as the ratio of adult males to sexually active females. Then, we included in the analyses age and mass as proxies of condition and status, as well as population size to understand whether ARTs are density‐dependent. Last, we measured for 83 randomly caught males (out of the 244; 66 were identified as roamers and 17 as territorials the month preceding these analyses), their body length, testes weight and the percentage of body mass testes occupied (i.e. the gonadosomatic index, GSI, see Supporting Information; Tomkins & Simmons, 2002), because differences in these physiological aspects would suggest a different allocation strategy towards costly reproductive traits. We counted sperm using a modified version of the procedure described by Wang (2002). Specifically, the epididymis was sliced in a phosphate buffer solution, the sperm suspension was incubated in a thermomixer for 10 min at 40°C to kill the sperm, and 10 mL of 1:20 or 1:40 diluted sperm suspension was used accordingly to get a sperm count of around 3–10 per square in a Burker chamber. Then, sperm numbers were counted twice under a microscope at 40× with a PH2 filter in 25 squares.

### 2.6 Antenna data and statistical analyses

In our independent populations, high‐resolution RFID antennas were fitted at the entrance of each nest (AniLoc, FBI Systems GmbH, Germany), and automatically recorded the time each mouse spent in every nest (König et al., 2015). Data were collected using the OLCUS IDE programme (FBI Systems GmbH, Germany).

All analyses, including the analysis of antenna data, were performed using R version 4.2.2.

### 2.7 Repeatability and modelling

To determine how much variation in males' choice of being territorial or roamer was due to differences between individuals, we calculated the repeatability of monthly tactic choice using the R package rptR (Stoffel et al., 2017). A relatively high estimate (mean estimate for behaviour is ~37%, Bell et al., 2009) would indicate that some males are consistently more likely to be territorial/roamer compared to conspecifics. Males with only one observation were excluded (we ended with a sample size of *N* = 199) and we assumed a binomial error structure. We included male identity as a random effect and age as a fixed effect to exclude its contribution to our estimate. The permutations and bootstrapping were set to 1000, and confidence intervals at 95%.

To correlate ARTs with reproductive success we regressed the four fitness metrics described in 2.4 (in four models) with the ratio of the times each male was caught as roamer to the total times been caught (formula a) for males with all monthly observations (*N*_males_ = 104). We first modelled the probability of reproduction as a 0/1 response (0 = no reproduction; 1 = reproduction) using a logistic regression (R package stats). Then, we modelled fitness (the individual number of offspring) using a two‐part zero‐altered negative binomial model ($\frac{R}{R+T}$ was included a predictor for values >0 and for the binomial part; pscl package, Zeileis et al., 2008), because many males did not reproduce and to correct for overdispersion. Last, relative fitness and fitness/age were response variables in two generalized linear models (R package stats) assuming a zero‐truncated gamma distribution. Importantly, the two latter metrics were zero‐inflated but we excluded the zeros since we considered them (the zeros) for the probability of reproduction and absolute fitness.

We used a generalized mixed‐effects model (glmm; R package lme4, Bates et al., 2015) with monthly tactic as a binomial response (territorial/roamer, 727 observations from *N*_males_ = 244) to identify the correlates of ARTs. To select the best predictors from a set of candidates (these predictors were chosen because they are consistently described to affect the expression of ARTs, see Introduction), we used the AICc (Table S1). Specifically, we explored if interacting mass with age has explanatory power, whether the OSR should be used or the number of sexually active females and of adult males as individual variables instead, if population size and the number of subadults increase the explanatory power of a model (detailed explanation in Table S1). Ultimately, our model included mass and age to test if status or age correlated with tactic, respectively, the OSR (adult males/sexually active females) to test if mate availability affected ARTs and population size (scaled, i.e. subtracted the mean and divide by the standard deviation) to test if ARTs were density‐dependent. Random effects included individual ID nested in enclosure ID. The best model performed better than a null model with only an intercept (*p* < 0.001).

Last, we used a multi‐state Markov model (msm: R package msm, Jackson, 2011) to explore the transition probabilities between ARTs, and the respective hazard ratios (Table S2; Figure S5), as a function of covariates for the 244 males. Transition probabilities for each tactic were modelled with 95% confidence intervals, accounting for individual identity and age. We allowed males to freely transition between tactics to match observational data (Figure S3), and death was the absorbing state. We used the same covariates as above in the glmm_s_ to predict transition intensities (Table S2). Age, OSR and population size predicted the transitions between territoriality to roaming and between territoriality to death. We assumed this because we expected territorials to get displaced/die with increasing age, as an indication of decreased competitive ability, and to be more likely to get displaced/die as intrasexual competition (higher OSR and population size) increases. Age, OSR and population size were also placed on the transition between roaming to death because we expected older roamers to be less competitive, and an increased competitive environment to increase death rates. Last, mass, OSR and population size modelled the transition between roaming to territoriality, assuming roamers that increase in weight, or experience less competition, will attempt to claim a nest‐box.

### 2.8 Intrinsic correlates

To compare the physiological traits between ARTs, we used a t‐test for comparing the mass between tactics per month, the testes weight and body length (both measured only once when animals were randomly removed from the populations and sacrificed) since they all followed a Gaussian distribution; and a Wilcoxon rank‐sum test for the testes to body mass ratio (GSI) and sperm counts because they did not (again both measured only once, when animals were randomly removed from the populations and sacrificed). Importantly, for the GSI, we ran an ANCOVA (Tomkins & Simmons, 2002) to explore the influence of monthly tactic on testes weight by controlling for the effect of mass, thus testing for a homogeneity of regression slopes between monthly tactic and mass (for details see Supporting Information).

### 2.9 Ethics statement

All animals were handled and procedures were carried out according to national guidelines. Procedures are approved under licences V244‐12767/2019 and V244‐31223/2019(62–5/19) by the Ministerium für Energiewende, Landwirtschaftliche Räume und Umwelt, Kiel. Keeping of mice was approved and is regularly controlled by the Veterinäramt Plön under permit: 1401‐144/PLÖ‐004697.

## 3 RESULTS

In our validation experiment using antenna data, a clear differentiation among males we had previously classified as territorials or roamers emerged: territorials spent most of their time in nest‐boxes while roamers were outside and only ‘visited’ nest‐boxes for a fraction of their total time (Wilcoxon rank‐sum tests, Figure 2: left graph *p* < 0.001; right graph *p* < 0.01). These data therefore reflect more detailed nest‐box use of males following different tactics. Consequently, we used males' location during each monitoring to describe the monthly tactic since it reflects activity within and outside the nest‐boxes.

Tactic was moderately repeatable (*R* = 0.18, CI = 0.067–0.3, *p* < 0.001). From the 199 (out of 244 available; Figure 1) males that were tested for repeatability, 9 were always territorial, 61 always roamers and 129 switched at least once. From the latter, 24% (31 males) switched more than once between territoriality and roaming (Figure S3).

**FIGURE 3:**
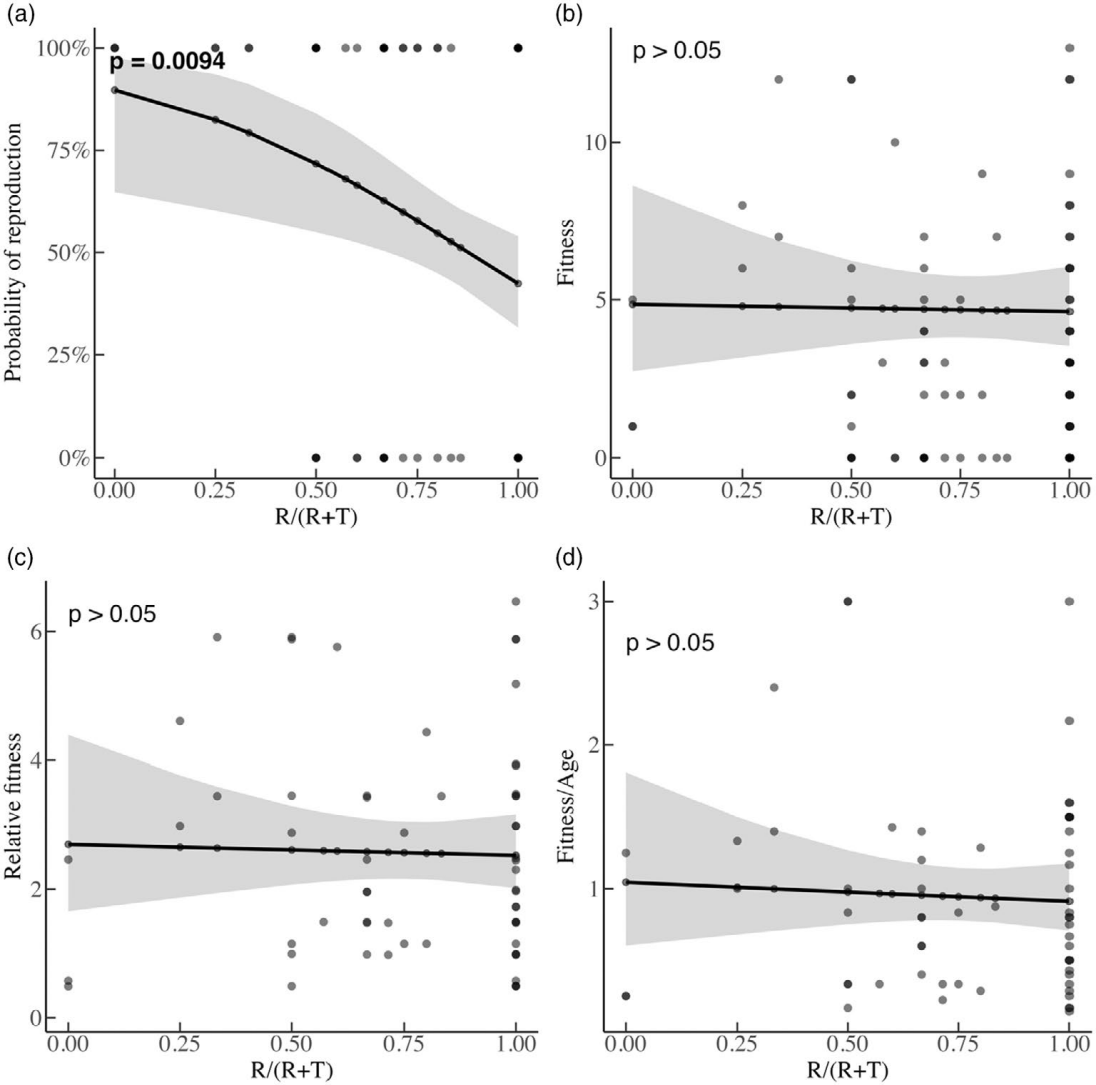
The effect of $\frac{R}{R+T}$ (0 being always territorial and 1 always roamer) on the (a) probability of reproduction; (b) the number of offspring; (c) relative fitness; (d) fitness/age. The line indicates the predicted effect by varying the respective focal variable. The shaded area represents the 95% CI. Black dots indicate overlapping datapoints and grey single.

The distribution of fitness among males for which we had all observations (104 out of the 244 available; Figure 1) was highly skewed with half not reproducing, and with reproducing males gaining between 1 and 13 offspring (mean = 2.7 ± 3.6; Figure S4). We found that males located more often as roamers (higher $\frac{R}{R+T}$ values) were less likely to ever reproduce (logistic & two‐part/hurdle model: *β* = −2.4, SE = 0.92, *p* = 0.009; Figure 3). However, $\frac{R}{R+T}$ did not predict fitness for the count part of the hurdle model (*β* = −0.04, SE = 0.20, *p* > 0.05), that is mean fitness did not change based on lifetime ART choices. When testing for the effect of $\frac{R}{R+T}$ on relative fitness and fitness/age, we again found no significance (relative fitness GLM: *β* = −0.06, SE = 0.30, *p* > 0.05; fitness/age GLM: *β* = −0.12, SE = 0.33, *p* > 0.05). Importantly, roamers sired offspring (61/104 males were always roamers and 22 of these 61 reproduced successfully) at some point during their life.

Focusing on all available males (244), that is adding to the 104 above those we had missing observations (140), did not alter these results: out of the 244, 19 were always territorial, 78 always roamers and 147 switched tactics at least once. The average fitness did not differ significantly between the categories as territorials had 2.47 ± 2.99 (mean & standard error) offspring, roamers 1.91 ± 3.16 and switchers 2.45 ± 3.64.

**FIGURE 4:**
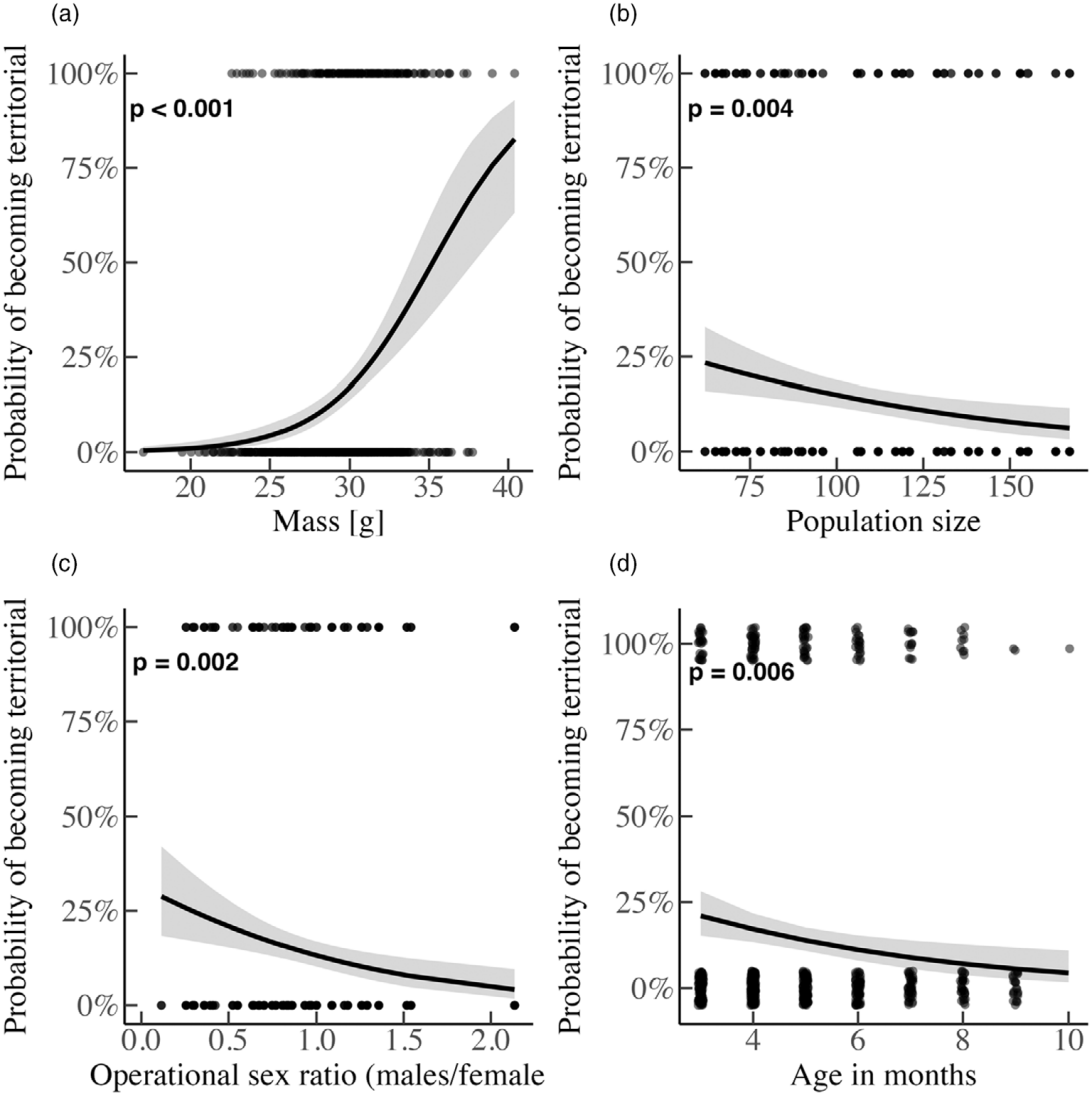
How (a) mass, (b) population size, (c) OSR and (d) age affect the probability of being territorial. The line indicates the predicted effect by varying the respective focal variable and by setting all the other covariates to their mean. The shaded area represents the 95% CI. Black dots indicate overlapping datapoints and grey single.

The odds of territoriality increased by almost 35% (glmm: 95% CI = [1.22, 1.50], *p* < 0.001; Figure 4a; Table S3) with an increasing mass, and one unit increase in age (i.e. 1 month) elevated the probability of roaming by 22% (95% CI = [0.65, 0.93], *p* = 0.006; Figure 4d). Increasing population size was associated with a drop of 36% in the probability of territoriality (95% CI = [0.47, 0.87], *p* = 0.004; Figure 4b). Last, a male‐biased OSR correlated with a 67% higher probability of roaming (95% CI = [0.17, 0.67], *p* = 0.002; Figure 4c; OSR: 0.11–2.13).

**FIGURE 5:**
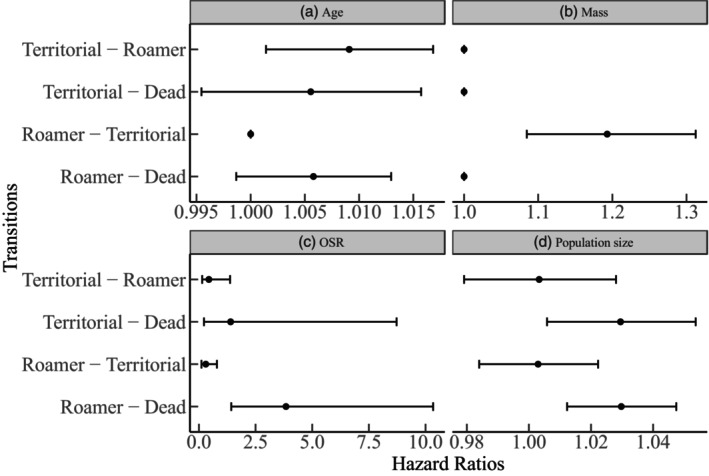
Hazard ratios of the covariates placed on each transition (dots without CIs are covariates not placed on the respective transition). The values represent how the instantaneous risk of making a particular transition is modified by the respective covariate (covariate shown on top).

Similar results were obtained from the multi‐state analysis (Figure S5). On average, the expected total length of stay in each state was ~55 days as a territorial and ~150 as a roamer, while a territorial remained so for ~20 consecutive days and a roamer as a roamer for ~65 days. ARTs also differed in baseline survival rates (with all covariates at their mean; Table S2). Specifically, territorials had a higher death probability after 30, 60 and 180 days compared to roamers (see Table S2). The covariates also affected the tactics differently. A higher OSR correlated with almost four times the risk of death for roamers (msm: 95% CI = 1.42–10.33) and with an increased risk of death for territorials (~40% higher, 95% CI = 0.22–8.7; Table S2; Figure 5c). Also, for each one‐gram increase in weight, a roamer was ~20% (95% CI = 1.08–1.31; Figure 5b) more likely to become territorial. In contrast, age and population size did not significantly (statistically or biologically; Figure 5a,d) affect transition rates between states.

**FIGURE 6:**
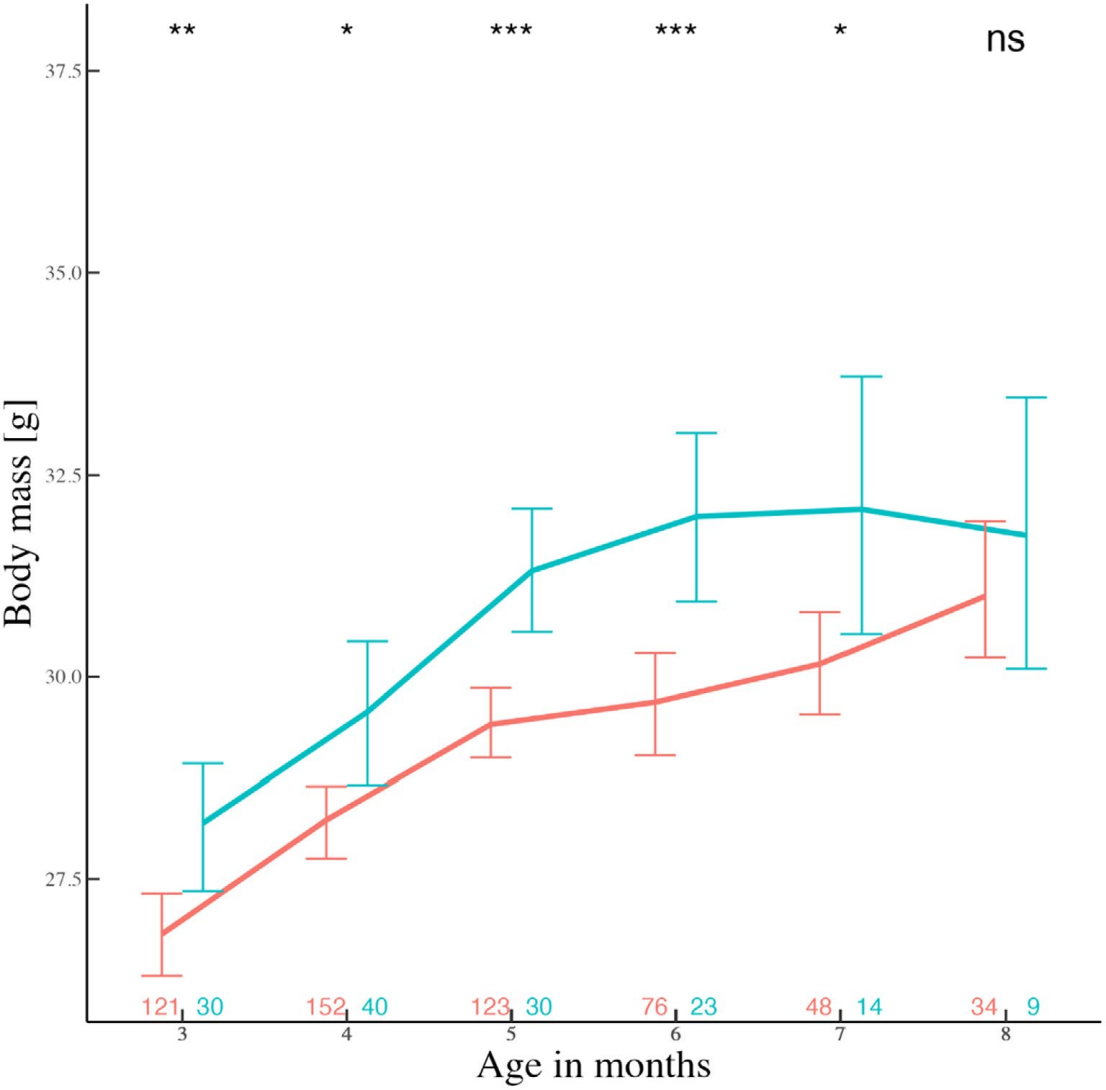
Growth curves and statistical significance (*p < 0.05, **p < 0.01, ***p < 0.001) when comparing the mean mass of ARTs across populations (red: roamers, blue: territorials) per month (monthly comparisons). Below each node, is the number of observations for the respective age. Colours match the respective tactic. All observations start from month three, when males become reproductively active. Error bars show the SEs.

Territorials were heavier than roamers except at an age of 8 months (Figure 6, but note the drop in sample size) and had a higher overall mean mass [*t*(226) = 5.7, *p* < 0.001; ${\bar{x}}_{\mathrm{ter}}$ = 30.5 & ${\bar{x}}_{\mathrm{sn}}$ = 28.9]. The GSI differed between ARTs (*W* = 332.5, *p* = 0.01; Figure S6) and this was true even when using an ANCOVA with testes weight as a response, mass as a covariate and monthly tactic as a predictor. In contrast, sperm count did not differ between ARTs (*W* = 417, *p* = 0.1). There was also an overall significant positive correlation between the GSI and sperm count (rho = 0.32, *p* = 0.003; Figure S7). In contrast, our analyses showed no difference between male ARTs in body length or testes mass (Table S4).

## 4 DISCUSSION

Here, we first found the choice of male tactic to be repeatable despite the fact that males switched between tactics frequently. Adoption of a tactic was also correlated to social and individual factors, which also affected the transition rates between them. Last, territorial mice were more likely to reproduce but had lower GSI values.

In all monitorings, some nest‐boxes were unoccupied (pers. observations & antenna data). Consequently, either males patrol more than one nest‐box or some stay roamers despite the potential to acquire a nest‐box. This latter might seem contradictory, as it would seem beneficial for males to acquire nest‐boxes that females aggregate to. It might be that here, as in many species, territoriality requires an elevated competitive ability or energy expenditure to deter conspecifics (Ord, 2021) and can increase stress (Hunninck et al., 2020), mortality and injury risk. Indeed, testosterone levels are positively correlated with energetic demands (Schradin et al., 2009) which in turn are associated with maintaining a territory (Ord, 2021). Alternatively, if the quality of roamers is not adequate to attract females then a parasitic tactic can provide fitness benefits for those males (Luttbeg, 2004).

### 4.1 Repeatability

We tested if tactic choice was repeatable despite the fact that the repeatability of male mating behaviour has received little attention (Hoefler et al., 2009; but see Magellan & Magurran, 2007; Rushbrook et al., 2008). Our results showed moderate repeatability (Bell et al., 2009), as ~20% of the variation between males in their tactic choice was explained by consistent between‐individual differences. Consequently, we report ARTs in house mice to have a personality component; but they are also flexible since individuals switch (Figure S3), something reported across rodents and other mammals (Schradin et al., 2009; Schradin & Lindholm, 2011; Schradin & Yuen, 2011; Shuster et al., 2019; Wolff, 2008). In other words, across time, some males tend to switch tactics more than others that switch less frequently or not at all. Our findings contrast with Schradin et al. (2009), who reported that territorial striped mice rarely switch, but they agree with a damselfly study (*Calopteryx maculate*; Forsyth & Montgomerie, 1987), and with other observations across mammals (reviewed in Wolff, 2008) or fish (reviewed in Taborsky, 2008), where territorials can transition to a roaming/sneaking tactic.

### 4.2 Fitness of ARTs

We showed the fitness difference between ARTs to lie in successful reproduction rather than in relative or absolute fitness. The more times a male was a roamer, the lower its probability of ever reproducing. Nevertheless, since some roamers did reproduce, mean fitness estimates were similar between males following different tactics. Comparatively, resident and wanderer male voles (Shuster et al., 2019) have equal fitness, and ARTs have no fitness effect under intermediate population densities in striped mice (Schradin & Lindholm, 2011). Therefore, our results suggest that not all males perform the most successful behaviour simultaneously (Brockmann, 2002). Ultimately, frequency‐dependent selection could create a pattern similar to ours through fluctuations due to breeding season or population size (Schradin & Lindholm, 2011), as shown in other species (e.g. negative frequency‐dependent selection in *Xiphophorus multilineatus* fish, Rios‐Cardenas et al., 2018).

Intense intersexual competition precedes the evolution of ARTs (Shuster & Wade, 2003) and here half of the males for which we had all observations never reproduced (Figure S4). While the fitness effect of ARTs in our populations lay in the probability of successful reproduction, the variance in reproductive success was higher for males adopting a roaming tactic most often, but the mean was equal (Figure 3). In other words, including males that never reproduce can shift the fitness consequences of ARTs (Brockmann, 2008; Shuster, 2011; Shuster et al., 2019). Equal mean fitness could be achieved because inside the enclosures (Porwal et al., 2023), as well as in natural populations, extra‐pair paternity is high (Schradin et al., 2010; Schradin & Lindholm, 2011) and roamers contribute towards it (Schradin et al., 2010). While the driver of extra‐pair paternity remains unknown (Schradin & Lindholm, 2011), it could be active female choice for roamers (Lehnert et al., 2017), coercion from roamers or both. Alternatively, the absence of predation and immigration in the enclosures might affect fitness across tactics, especially since predation risk should affect which tactic males choose (e.g. in crickets Torsekar & Balakrishnan, 2020).

### 4.3 Correlates and transitions rates of ARTs

Male ARTs are often dependent on condition or status (Brockmann, 2001; Gross, 1996; Johnson & Brockmann, 2012; Shuster & Wade, 2003), differentially expressed under variable population sizes (Radwan, 1993; Radwan et al., 2014) and affected by female availability and the OSR (Eberle & Kappeler, 2004; Wolff, 2008). Here, a higher weight increased the odds of a male being territorial (Figure 4a), predicted a transition between roaming to territoriality (Figure 5), and territorials were consistently heavier than roamers (Figure 6). Therefore, heavier males, as in other rodent species (Schradin et al., 2009), control important resources such as a nest‐box in our case. Body weight, which probably reflects competitive ability, condition and strength, explains variation in tactic choice and tactic transitioning. Our findings hint that status‐dependent selection acts in our populations despite the fact that tactic choice did not predict relative fitness (Schradin & Lindholm, 2011). Importantly, the observed mass difference between ARTs was minimized from month 8 (onwards), probably due to a low number of territorials.

If territorial acquisition is associated with increased competition (to defend females or pups, Schradin et al., 2009), then older males might be worse at deterring conspecifics (Rathke et al., 2017) and defending their territory (Forsyth & Montgomerie, 1987). However, an increasing age did not predict an immediate transition to roaming/death, but it did associate with increased odds of roaming (Figure 5a). In other words, the probability of roaming increases with age, possibly because young males first become roamers, which also means that young males cannot easily transition to territoriality unless they grow large enough (as discussed earlier). Moreover, since young males usually first become roamers, male–male competition for territories is not strong enough to produce a higher risk of death for either tactic, which explains why the transition from either tactic to death was statistically non‐significant. Importantly, since our observations are restricted up to a maximum age of 11 months, future studies could specifically attempt to understand how ARTs develop in the later stages of life (but note that 10 months is at the upper end of age naturally occurring mice are reported to reach: Berry & Jakobson, 1971; Morgan & Bellamy, 1975).

~25% of territorials that switched to roaming at any point died within a month of doing so, and mortality risk was consistently higher for territorials (Table S2). Therefore, a transition from territory‐holding to roaming possibly reflects the effect of aging on body condition or elevated stress levels, which increase mortality. In other words, our analyses revealed that some roamers stay as such throughout their life and that some territorials cannot easily cope with losing their nest‐box (as described above). However, a lot of males switched (Figure S3), an indication that some individuals are more plastic than others. In turn, this might suggest the existence of three tactics (territorials, roamers & switchers), something that future studies could address.

A male‐skewed OSR associated with a decreased probability of territoriality and of survival in both tactics. Here, it might be that as male–male competition increases (more adult males in the population; hence a higher OSR), available territories get depleted more quickly, rendering roaming inevitable, and the death rate elevates too, with the effect being (~three times) stronger among roamers. Therefore, we suggest that the costs of roaming mainly stem from intense intrasexual competition, which intensifies as roamers interfere with more males (Jirotkul, 1999a). We believe that the comparatively smaller effect size of an increasing OSR (which results from more adult males being present) on territorials is because these males are the heavier and most competitive at any point, thus can cope with intense competition. If a higher OSR is instead due to less sexually active females, this could select for male roaming as a way to locate females. However, our results showed no transition rate from territoriality to roaming as the OSR increased; thus, changes in the OSR mainly reflect fluctuations in male–male competition. Comparatively, in mouse lemurs, switching between tactics depends on short‐scaled variation in aspects such as fertilization probability, the number of alternative mating opportunities and the operational sex ratio (Eberle & Kappeler, 2004).

An increasing population size correlated with a higher likelihood of roaming but did not affect immediate transition rates. This is because a higher population size depletes the available nest‐boxes and new males that enter the population, as roamers cannot displace territorials. Comparatively, density affects ARTs across taxa (Brockmann, 2008; Forchhammer & Boomsma, 1998; Karkarey et al., 2017; Wolff, 2008) but only when the number of males exceeds that of territories (Forsyth & Montgomerie, 1987). Indeed, increased density intensifies male–male competition (in our study a higher population size predicts a higher probability of bitemarks at the population level; results not shown), and this is shown experimentally too (Jirotkul, 1999b).

Overall, territorials and roamers that face the same amount of competition and have similar age and weight differ in their lifespan, with the former suffering from a higher death rate, possibly due to the condition‐dependent and physiological costs associated with maintaining a territory (Ord, 2021). However, as male–male competition increases, roamers are dying at higher rates (Figure 5), which means that density, age, male–male competition and status (weight) affect the odds of occupying a tactic; but only the latter two interact to affect transition rates (Figure 5).

### 4.4 Physiological correlates of ARTs

Theoretically, males should adjust ejaculate size in respect to the risk and intensity of sperm competition (Montgomerie & Fitzpatrick, 2019) and roamers should invest more in sperm traits (Dougherty et al., 2022; Kustra & Alonzo, 2020). In our study, the GSI was higher for roamers despite the mean testes' weight and sperm count not differing between ARTs, the latter in accordance with recent reviews (Dougherty et al., 2022; Kustra & Alonzo, 2020; but most vertebrate studies come from fish). Our results suggest that roamers increase ejaculate size or frequency (Montgomerie & Fitzpatrick, 2019), similar to sneaker fish that have larger testes than non‐sneakers (Dougherty et al., 2022; Montgomerie & Fitzpatrick, 2019). Territorials might be unable to afford the energetic requirements needed to maintain increased testis size as they allocate energy into courtship and defending their territory (Montgomerie & Fitzpatrick, 2019).

Sperm count and animal body size did not differ between male ARTs. In striped mice, males of ARTs invest equally in sperm (Schradin et al., 2012) and across taxa, there is no significant difference in sperm traits (sperm density, volume or number) (Dougherty et al., 2022; Kustra & Alonzo, 2020). In our study, it might be that sperm quality, seminal fluid components or hormones (Kustra & Alonzo, 2020), rather than sperm quantity, might be under selection, something future studies should test. Male ARTs could differ in sperm allocation or seminal fluid composition and not sperm expenditure (Dougherty et al., 2022), so that roamers allocate more sperm or seminal fluids per copulation to increase their fertilization probability or decrease dominant males' sperm performance, respectively (Dougherty et al., 2022). Alternatively, it might be that roamers do not invest more in sperm due to trade‐offs (e.g. energetic trade‐offs; reviewed in Kustra & Alonzo, 2020).

## 5 CONCLUSIONS

Overall, here we provide robust evidence that ARTs in male *Mus musculus* exist and that, while territorial males have a higher probability of reproduction, ARTs have equal mean fitness. We further show ARTs to vary considerably within individual lifespan in response to social (OSR and population size) and intrinsic (mass and age) features, and to display differences in testes size but not in sperm number. Crucially, our results suggest that male mice display phenotypically plastic ARTs, which can nevertheless be explained by between‐individual differences over time (repeatability). Whether sexual selection acts more within tactics than between, as a result of the higher variance in fitness within males found mostly as roamers, is a topic that should be explored by future studies.

## AUTHOR CONTRIBUTIONS

F. Darmis performed all analyses. F. Darmis and A. Guenther contributed to conceptualization. A. Vezyrakis contributed to data collection and analysis of the antenna data. A. Guenther conceived and supervised the study design. F. Darmis wrote the first draft, and all authors revised the subsequent versions.

## CONFLICT OF INTEREST STATEMENT

The authors declare that the research was conducted in the absence of any commercial or financial relationships that could be construed as a potential conflict of interest.

## Supporting information


**Table S1:** AICc selection table for MS models.
**Table S2:** Probability of transitioning between territoriality and roaming, and state‐related survival (in grey), across various periods with covariates set to their mean.
**Table S3:** Results of the model 4 with the probability of being territorial as the response variable.
**Table S4:** Results of testing for correlations between ARTs and physiological measurements.
**Figure S1:** Home ranges of males and females that mated, as well as of their offspring, in an independent experiment run in 2017 in one of our seminatural enclosures (Taken from Krebs‐Wheaton R. 2024; https://macau.uni‐kiel.de/receive/diss_mods_00022305?lang=en).
**Figure S2:** An example of a semi‐natural enclosure and the setup we used in our long‐term experiments of male ARTs in house mice. Houses/Nest‐boxes (orange arrows) are indicated and food was distributed equally across the room and provided *ad libitum*.
**Figure S3:** The variation in the choice of tactic within and between males: The ID of individuals is on the y‐axis while the age in months is on the x‐axis.
**Figure S4:** Left plot: The number of males that did not (indicated at 0; *N* = 50) and did (indicated at 1; *N* = 54) reproduce; Right plot: The number of offspring of males that reproduce, with count being how many males had the respective number of offspring.
**Figure S5:** The multi‐state model, with the associated Q matrix, shown diagrammatically.
**Figure S6:** Violin plots showing the variation in roamers (*n* = 66) and territorials (*n* = 17) in respect to traits thought to relate to sperm competition, i.e., the GSI (*y*‐axis) and the number of sperm (*x*‐axis).
**Figure S7:** The correlation between the sperm count/mL and the GSI for all males of both tactics (i.e., at male population level).
**Figure S8:** How population size (a) and OSR (b) changed in the course of the study in the four semi‐natural enclosures.

## Data Availability

The data to replicate all analyses are available here: https://doi.org/10.5061/dryad.s7h44j1dm (Darmis et al., 2025).
