# Supplementary material for: Male reproductive tactics in house mice: Consistent individual differences, intrinsic factors and density effects
Source: J Anim Ecol. 2025 Apr 12;94(6):1244–58. doi: 10.1111/1365-2656.70039 (PMC12134446; doi:10.1111/1365-2656.70039)
Supplement: Supplementary file 1 — Table S1: AICc selection table for MS models. Table S2: Probability of transitioning between territoriality and roaming, and state‐related survival (in grey), across various periods with covariates set to their mean. Table S3: Results of the model 4 with the probability of being territorial as the response variable. Table S4: Results of testing for correlations between ARTs and physiological measurements. Figure S1: Home ranges of males and females that mated, as well as of their offspring, in an independent experiment run in 2017 in one of our seminatural enclosures (Taken from Krebs‐Wheaton R. 2024; https://macau.uni‐kiel.de/receive/diss_mods_00022305?lang=en). Figure S2: An example of a semi‐natural enclosure and the setup we used in our long‐term experiments of male ARTs in house mice. Houses/Nest‐boxes (orange arrows) are indicated and food was distributed equally across the room and provided ad libitum. Figure S3: The variation in the choice of tactic within and between males: The ID of individuals is on the y‐axis while the age in months is on the x‐axis. Figure S4: Left plot: The number of males that did not (indicated at 0; N = 50) and did (indicated at 1; N = 54) reproduce; Right plot: The number of offspring of males that reproduce, with count being how many males had the respective number of offspring. Figure S5: The multi‐state model, with the associated Q matrix, shown diagrammatically. Figure S6: Violin plots showing the variation in roamers (n = 66) and territorials (n = 17) in respect to traits thought to relate to sperm competition, i.e., the GSI (y‐axis) and the number of sperm (x‐axis). Figure S7: The correlation between the sperm count/mL and the GSI for all males of both tactics (i.e., at male population level). Figure S8: How population size (a) and OSR (b) changed in the course of the study in the four semi‐natural enclosures. [file JANE-94-1244-s001.docx]

**Supplementary material**

**Use of the GSI**

After comparing the GSI between tactics, we ran an analysis of covariance, as suggested by Tomkins & Simmons (Tomkins & Simmons 2002), to explore the influence of monthly tactic on testes weight whilst controlling the effect of mass and further confirm if there are differences in testes weight between ARTs. A homogeneity of regression slopes indicated that the interaction term (monthly tactic*mass) was not statistically significant (F (1, 79) = 2.65, p = 0.1), thus we removed this interaction: ultimately, after adjustment for mass, there was a statistically significant difference in testes' weight between the groups (F (1, 80) = 5.306, p < 0.024), and for that reason we retained the GSI in our analysis.

**Table 1**

AICc selection table for MS models. Individual identity was specified as a random effect nested in enclosure for all models. In model 2 we experienced converge problems. In model 4, population size was scaled to avoid convergence warnings. Here, model 4 is selected as it displays the lowest AICc value with the highest AICc weight.

Model 1 used as predictors mass, the number of adult males and of active females as well as age as predictors; model 2 has the same structure as model 1 but also explores if the interaction between mass and age improves the fit of our model in the data since, at least theoretically, older males should be less heavy and less competitive and *vice versa*; then, model 3 was fitted to explore if using the OSR is a better fit to the data than using individual metrics of adult males and active females. We replaced these two metrics with the OSR, which is just the fraction of the former to the latter; model 4 is a modification of model 3 as it just adds population size, a well described predictor of variation in ART expression, to test if it improves the fit of our model in the data; model 5 was fitted as an alternative to models 1,3&4 that includes the total number of males and of females instead of the population size, the OSR or the individual metrics of adult males and reproductively active females.

| Model | Model parameters | AICcWt | AICc value |
| --- | --- | --- | --- |
| Model 1 | MS^1^ ~ mass^2^ + males^3^ + active females^4^ + MM^5^ | 0.19 | 685.45 |
| Model 2 | MS ~ mass*MM + males + active females | 0.07 | 687.52 |
| Model 3 | mass + MM + OSR^6^ | 0.02 | 689.54 |
| Model 4 | mass + scale (pop size)^7^ + MM + OSR | 0.71 | 682.74 |
| Model 5 | mass + pop males^8^ + pop females^9^ + MM | 0.01 | 690.49 |

^1^The monthly strategy implemented (territorial or roamer); ^2^The mass of the individual; ^3^The number of sexually active (i.e., adult) males; ^4^The number of sexually active females; ^5^Age; ^6^Operational sex ratio, as the number of adult males to sexually active females; ^7^Population size scaled (i.e., subtracted by mean and divided by its standard deviation); ^8^The total number of males in the population; ^9^The total number of females in the population.

**Table 2**

Probability of transitioning between territoriality and roaming, and state-related survival (in grey), across various periods with covariates set to their mean. Transition probabilities are estimated from a multi-state Markov model fitted N=228 males. The model results (and the transition intensity matrix) are shown diagrammatically in-text (Fig. 4) and below (Figure S4). Essentially, each number represents the probability of completing the specified transition at the respective time (shown in the columns).

| Transition | 1-month probability (95% CI) | 2-month probability (95% CI) | 6-month probability (95% CI) |
| --- | --- | --- | --- |
| Territorial 🡪 Territorial | 0.294 (0.187, 0.401) | 0.166 (0.117, 0.239) | 0.111 (0.073, 0.141) |
| Territorial 🡪 Roamer | 0.518 (0.379, 0.650) | 0.545 (0.392, 0.619) | 0.429 (0.303, 0.499) |
| Territorial 🡪 Dead | 0.189 (0.129, 0.312) | 0.289 (0.227, 0.453) | 0.460 (0.391, 0.609) |
| Roamer 🡪 Roamer | 0.738 (0.676, 0.778) | 0.621 (0.538, 0.670) | 0.469 (0.358, 0.537) |
| Roamer 🡪 Territorial | 0.154 (0.110, 0.199) | 0.153 (0.108, 0.195) | 0.121 (0.078, 0.152) |
| Roamer 🡪 Dead | 0.108 (0.084, 0.181) | 0.226 (0.184, 0.327) | 0.411 (0.344, 0.550) |

**Table 3**

Results of the model 4 with the probability of being territorial as the response variable. Estimate measures the odds of becoming territorial compared to the reference category (i.e., roamer) and is given in log-scale. “SE” indicates the standard error and CI shows the 95% confidence intervals. Statistically significant results are indicated with bold.

| Coefficients | Estimate (Log-Odds) | SE | Confidence interval | p-value |
| --- | --- | --- | --- | --- |
| Intercept | -8.35 | 1.39 | -11.07 – -5.62 | **<0.001** |
| Mass | 0.30 | 0.05 | 0.20 – 0.40 | **<0.001** |
| Population size | -0.45 | 0.15 | -0.75 – -0.14 | **0.004** |
| Age | -0.25 | 0.09 | -0.43 – -0.07 | **0.006** |
| OSR (♂/♀) | -1.10 | 0.36 | -1.80 – -0.41 | **0.002** |

**Table 4**

Results of testing for correlations between ARTs and physiological measurements. The result of the respective statistical test is shown, along with the mean or median per ART. Statistically significant results are indicated with bold.

| Physiological measurement | Type of test | Territorials | Roamers | p-value |
| --- | --- | --- | --- | --- |
| Animal size (body length) | Two-sample t-test | 9.835294 cm | 9.660606 cm | 0.27 |
| Testes’ weight | Two-sample t-test | 0.1795882 g | 0.1945303 g | 0.19 |
| Testes weight to body mass (GSI) | Two-sample Wilcoxon test | 0.57 | 0.635 | **0.01** |
| Sperm count/mL | Two-sample Wilcoxon test | 1100 | 1270 | **0.10** |
| Mass | Two-sample t-test | 32.19471 g | 30.54212 g | **0.095** |

**
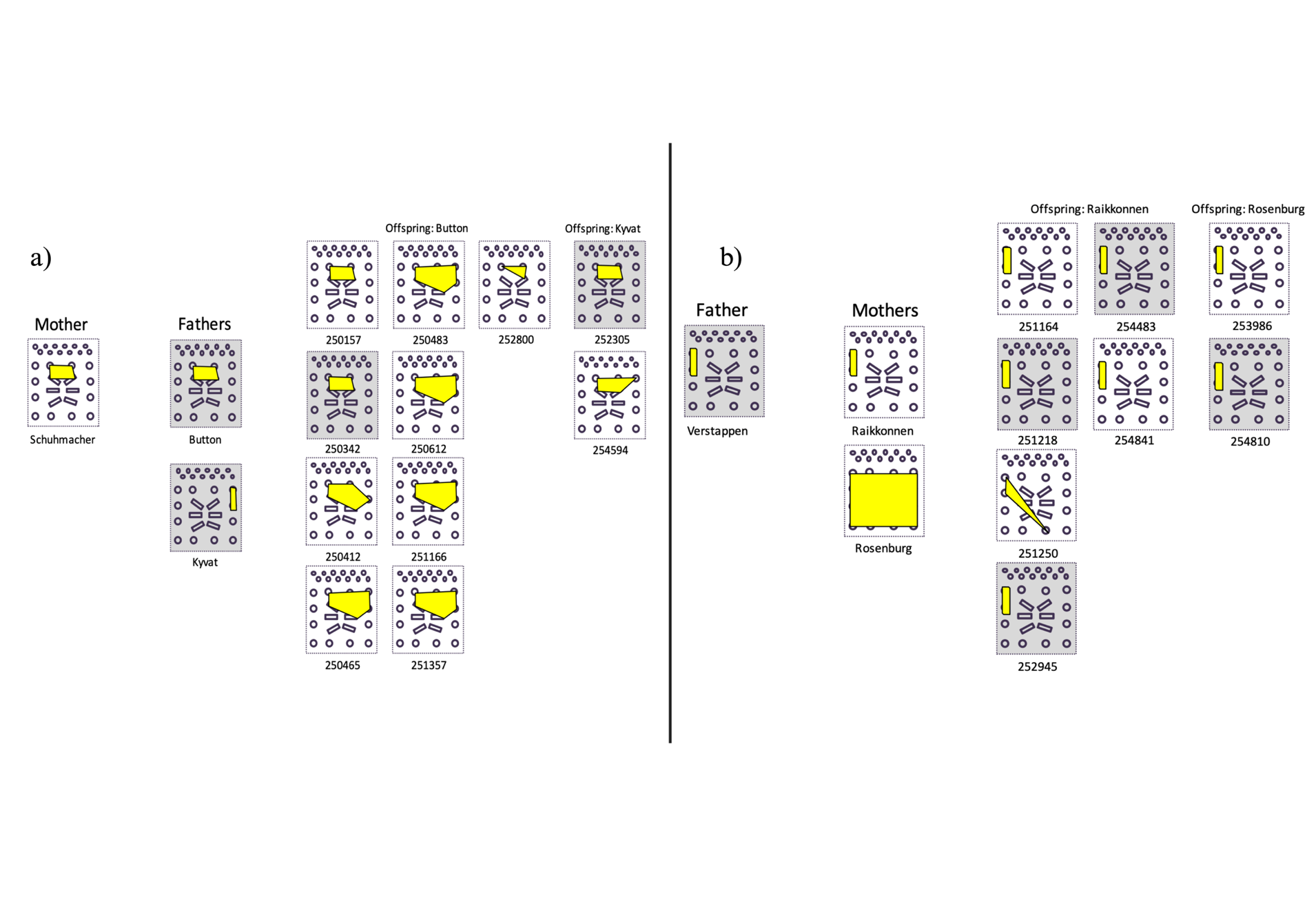
**

**Figure 1:** Home ranges of males and females that mated, as well as of their offspring, in an independent experiment run in 2017 in one of our seminatural enclosures (Taken from Krebs-Wheaton R. 2024; doi:<https://macau.uni-kiel.de/receive/diss_mods_00022305?lang=en>). Data show that mothers and fathers tend to overlap in territory use (indicated with yellow color):

a) Home ranges of the offspring of female mouse “Schuhmacher” and their respective fathers in enclosure A. Grey indicates that the offspring is male, white background indicates female mice. MCP indicates the area in which the mouse was found 95% of the time.

b) Home ranges of the offspring of male mouse “Verstappen” and their respective mothers in enclosure A. Grey indicates that the offspring is male, the white background indicates female mice. MCP indicates the area in which the mouse was found 95% of the time.


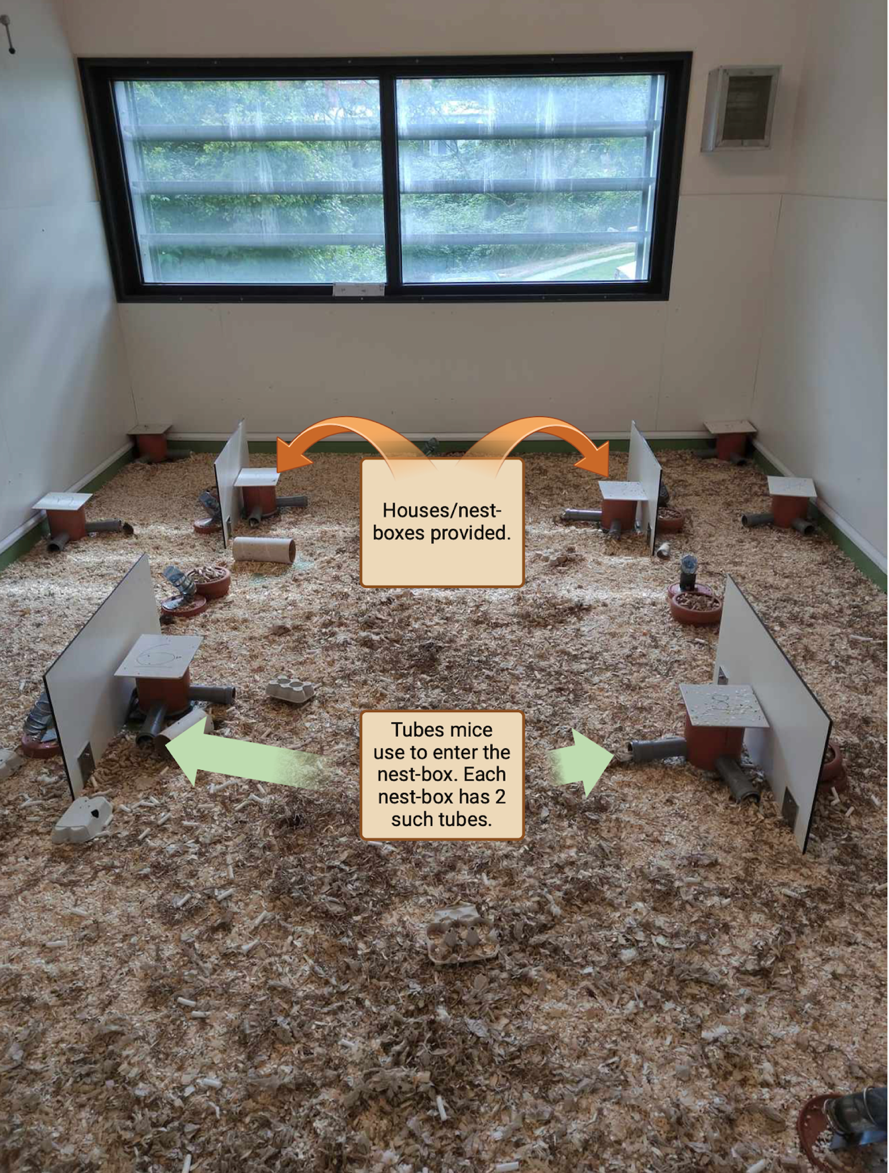


**Figure 2:** An example of a semi-natural enclosure and the setup we used in our long-term experiments of male ARTs in house mice. Houses/Nest-boxes (orange arrows) are indicated and food was distributed equally across the room and provided *ad libitum*. The procedure followed during our monthly monitorings, which led to the categorization of males to thw two ARTs (X and Y) was the following: At regular 4-week intervals, we entered the room, and as a consequence, mice behaved in either of three ways: (1) successfully attempted to enter the houses/nest-boxes; (2) unsuccessfully attempted to enter the houses/nest-boxes (got aggressively driven away by conspecifics, pers. observations); (3) or some did not attempt to enter and stayed outside. We then closed all entrances of the houses (green arrows) and caught all individuals inside the house, for which we measured their body mass (and consequently, because we did that on a monthly basis, we had age-relevant data). After taking out all available houses, we entered the room and caught the remaining individuals with traps, and followed the same procedure as before (i.e., measured their body mass). At last, all individuals were released back in the enclosure. Importantly, and because from the start of the experiment we had the IDs of the founders, as well as monthly data on the IDs of the offspring which we chipped, we could calculate exactly how many individuals we had each month in the population beforehand (hence we had data on population size and density) and compare that with the number of individuals we found in each successive month. Our design allowed males the possibility to flexibly switch their position per month, and as discussed before, we have validated this method of assignment of reproductive tactics (i.e., inside or outside houses) with the antenna data we describe in-text (details in Methods and Figure 1 in the main text).

**Figure 3:** The variation in the choice of tactic within and between males: The ID of individuals is on the y-axis while the age in months is on the x-axis. In blue are the males that follow a roaming tactic and orange those that were territory holders. All the males used in the analyses (but in fitness estimates) are presented here.

**Figure 4:** Left plot: The number of males that did not (indicated at 0; N=50) and did (indicated at 1; N=54) reproduce; Right plot: The number of offspring of males that reproduce, with count being how many males had the respective number of offspring.


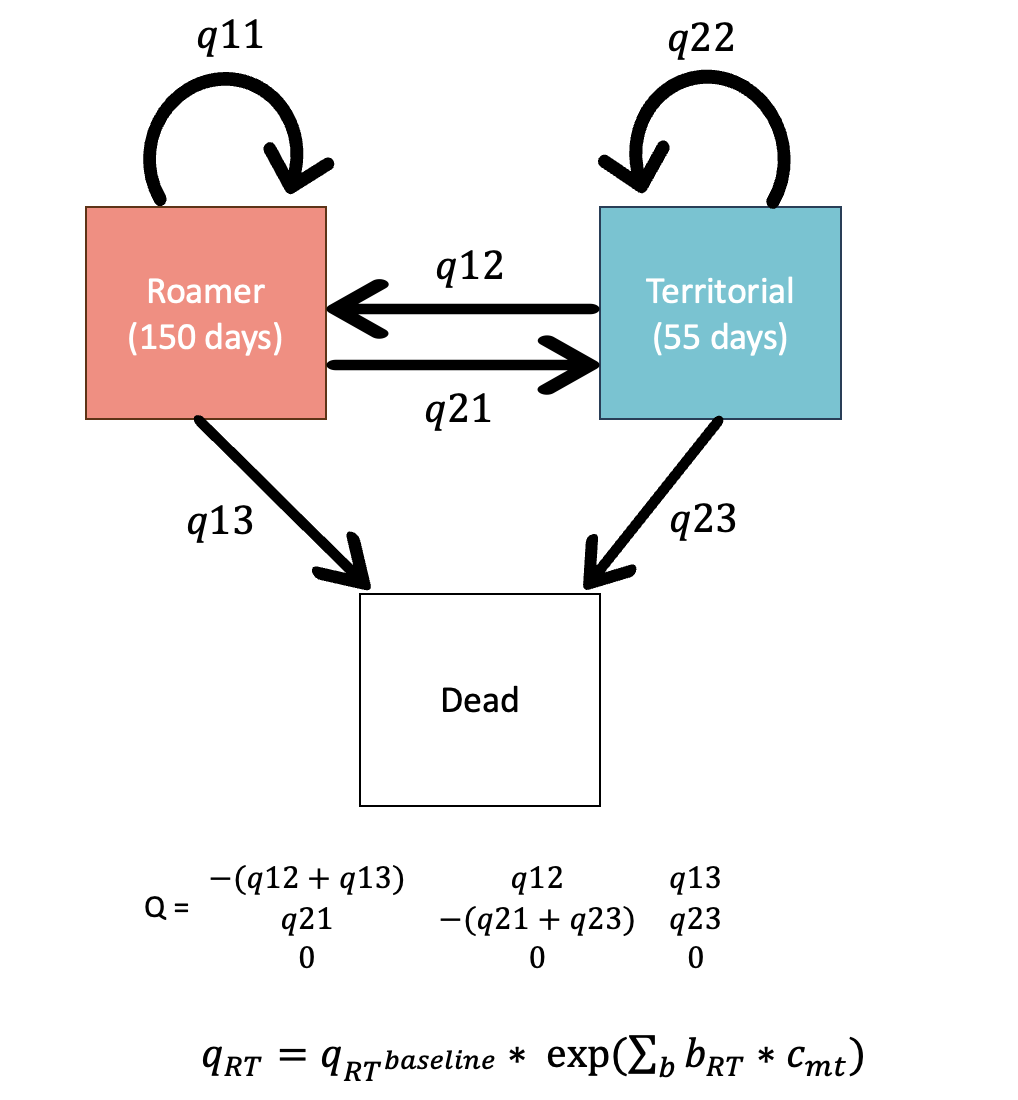


**Figure 5:** The multi-state model, with the associated Q matrix, shown diagrammatically. Inside each box, the median number of days a male spends in each state is shown. The formula calculates the transition rate  for each individual at a particular point in time, based on their specific covariate values at that time: each transition rate can be calculated by multiplying the baseline hazard ratio $(the rate when covariates are at their baseline values; \boldsymbol{q}_{\boldsymbol{RT}^{\boldsymbol{baseline}}}$) of the respective transition (*RT*, that is the transition from roaming to territoriality) with the exponentiated sum of the hazard ratio values associated with the **b-th** covariate of that transition ($\boldsymbol{b}_{\boldsymbol{RT}}$) times the value of that covariate for male **m** on time **t** (and this for all covariates placed on that transition; **Σ*_b_***). The equation is given at the end and is an example of the transition between roaming to territoriality.

**Figure 6:** Violin plots showing the variation in roamers (n = 66) and territorials (n = 17) in respect to traits thought to relate to sperm competition, i.e., the GSI (y-axis) and the number of sperm (x-axis). To detect differences between the two ARTs we compared the mean of both metrics of the two groups using a Wilcoxon rank sum test/Mann-Whitney U test for GSI and sperm count/mL. For the former, the comparison showed a significant difference between the ARTs. For the latter, the comparison was non-significant, with roamers having a mean sperm count of 1270 and territorials of 1100, a result that might indicate that a bigger sample size of territorials could yield statistical significance.


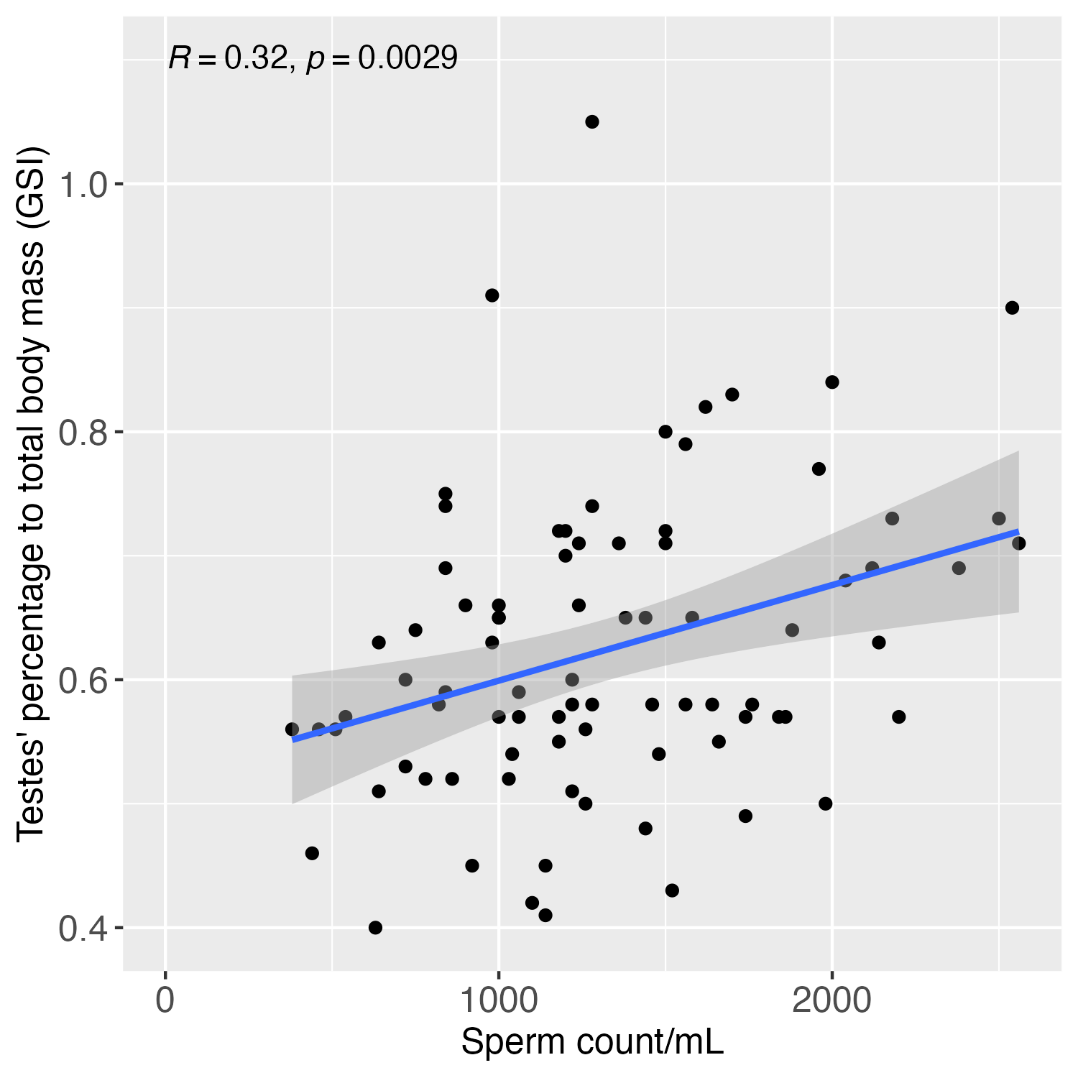


**Figure 7:** The correlation between the sperm count/mL and the GSI for all males of both tactics (i.e., at male population level).

**Figure 8:** How population size (a) and OSR (b) changed in the course of the study in the four semi-natural enclosures.
